# Editorial

**Published:** 1876-01

**Authors:** 


### Editorial.

### DISEASES OF THE GUMS AND TREATMENT.

This is an important subject and one to which more attention should be given than is usually bestowed upon it. It is one intimately connected with the every day practice of the dentist. It is one that involves not only the structure here mentioned, the gums and alveoli, in disease, but it effects the salvation of the teeth as well. The gums scarcely ever become diseased that the teeth do not more or less, (and frequently very seriously) suffer in consequence, and when the gums and alveoli both become diseased the teeth are always necessarily involved in diseases. In order to understand the pathological changes which take place in these structures, it is necessary to understand their anatomical and physiological peculiarities and their susceptibilities to disease producing influences. The gums are composed of a superficial layer, the mucous membrane, beneath which, is a highly vascular tissue. The membrane proper is largely endowed with exudation follicles both secreting and excretory follicles, the underlying vascular tissue is fibrous. This covers the alveoli and forms a margin which embraces the teeth beyond the termination of the alveolus, and is to a greater or less extent united to the necks of the teeth. The alveolus is the structure of bone underneath, which forms the sockets in which the roots of the teeth are imbedded. The gums are subject to various diseases and sympathize with disturbed conditions in other parts of the system. If the body is well nourished the gums partake of the general favorable condition. Good nutrition is an important matter.

Sometimes the vital forces are reduced to so low a point, the resistance to disease producing agents so enfeebled, through defective nutrition, that the mucous membrane, without any appreciable local influences, will assume a suppurating condition, aphthous patches will make their appearance in the mouth. This is too often attributed to local irritation merely. Then there are various susceptibilities, some constitutions resisting successfully the inroads of disease and others succumbing at the first attack. Every one finds cases in which the wonder is that the gums maintain their integrity as well as they do. In one instance the teeth may be invested with offensive accumulations in the mouth, full of old roots and in spite of all, the gums remain healthy. In another instance a single root may produce active disease which will involve remote parts. Why is this? It is plainly a result of difference of susceptibility.

It is often very important to correct defective nutrition. Good feeding will do as much to restore a healthy condition in the gums as in other structures of the system.

Local treatment will not avail without attention to the general systemic condition.

The gums are often starved—atrophied. Local treatment will not help that. We hear persons say "Oh, I have had my gums treated repeatedly and it has not benefited me in the least." Such treatment has only been partial and not based upon a thorough and far reaching diagnosis.

There is sometimes found a vascular enlargement of the gums accompanied by suppuration from the gums about the necks of the teeth, and a disintegration of the alveolus to a greater or less extent. The mucous membrane and the inner surface of the gums secrete an acrid, vitiated matter, which is very irritating. It is vitiated at first and becomes more so after it is discharged. As a result the gum becomes separated from the teeth and the alveolus dissolves away. The disintegration is also due partially to the presence of foreign substance beneath the margin of the gum. Though we may sometimes find a considerable deposit of calculus beneath the margin of the gum yet the gum remain comparatively healthy.

The exudation which I have mentioned is also sometimes observed on the external surface of the gum in the buccal or labial surface. This secretion indicates an effort of nature to dissolve away the obstructions or irritants. Frequently the alveolus will be dissolved away while the deposits of calculi, etc., remain and increase, encroaching even to the very end of the root. Sometimes nature completes a cure if there is a careful removal of all the particles of deposit, but generally it is necessary to employ agents to remedy systemic disorders. A gentleman related to me a case where the teeth became loose in this manner. He had removed all foreign deposit as he supposed. (It is a question whether this is generally done in a perfectly thorough manner. Instruments made for the purpose of removing such deposits are frequently ill adapted to a complete performance of the operation.) He found it necessary to cut away the free margin of the gums to induce healthy granulations. This operation is to be sometimes commended because it relieves the engorged vessels. Topical applications to reduce the circulation, may be made. The aim should be to force out the effete, poisoned blood and allow fresh, healthy blood to flow in. Dr. Watt suggested to me a new idea sometime ago, to manipulate the gums with the fingers, pressing out the vitiated blood and inducing a flow of healthy blood, where there is a tumefied, hypertrophied condition. It is remarkable how rapidly a cure is often affected under such treatment.

---

### GOODYEAR DENTAL VULCANITE CO.

The following from Johnston's Dental Miscellany, will doubtless be received with gratification by the readers of the Register.

Fear has been entertained, based mainly upon threats, that the Dental Vulcanite Co., would capture celluloid, but the remarks of the Court and the refusal to grant an injunction in this case, indicates that the ability of the Dental Vulcanite Co., is not equal to its desire. We are glad that a resting place is at last found in this matter.

"The Vulcanite Company recently commenced a suit against Dr. Eben M. Flagg, of this city, (New York) to compel him to take a license from them, or desist from the use of celluloid.

They asked the Court to grant an interlocutory or temporary injunction, restraining Dr. Flagg from the use of celluloid, pending the final decision of the case. This Judge Blatchford refuses to do, in the following decision.

### U. S. Circuit Court.

THE GOODYEAR DENTAL VULCANITE COMPANY AND OTHERS,

*vs.*

EBEN M. FLAGG.

BLATCHFORD, J.

I do not find that any decision has been made in regard to the Plaintiffs' patent which gives to it such a construction as necessarily includes the process and substance used by the Defendant. In the Gardiner case the Defendant did not compound india-rubber with iodine, and he employed heat to harden the rubber (Goodyear Dental Vulcanite Co., *vs.* Gardiner, 4 Fisher's Patent, Cases 224—231). In the Smith case the view of the Court was that the material to be used under the Plaintiffs' patent in carrying out the invention patented was to be india-rubber "and the compounds commonly employed therewith, reduced to a soft plastic condition, capable of vulcanization. and subsequently vulcanized." (Goodyear Dental Vulcanite Co., *vs.* Smith, 5 Official Gazette of Patent Office, 585.)

It appears from the description of the process used by the Defendant in this suit, that he does not use india-rubber or any substance capable of vulcanization; that the substance he uses is one which is rendered plastic by heat and is not hardened by heat, that heat is used in the process to soften the substance and render it plastic, and not to harden it, and that the substance, after being moulded, is hardened by being cooled. It is not sufficiently clear that this process is embraced in the claim of the Plaintiffs' patent to warrant the granting of an injunction, until one is awarded as the result of a decree for the Plaintiff on final hearing.

E. N. Dickenson and B. F. Lee,
For the Plaintiffs.

W. D. Shipman, C. A. Seward and E. L. Hamilton.
*Decision Dec. 7, 1875.* For the Defendant.

### MEETING OF OHIO STATE DENTAL SOCIETY.

The tenth annual meeting of this body was held Dec. 1st, 2d and 3d. There was about the usual number in attendance, and a decided interest was manifested in the proceedings. A number of papers was read, upon the subjects presented in the programme, and quite interesting discussions were had upon them. The time was very closely occupied, three sessions being held each day, except the last.

The Society decided to publish its transactions in a separate volume, and not in the Register as heretofore; this in some respects is an improvement, and perhaps in no particular, objectionable. This society is one of the best in the country, it transacts its business quietly, unostentatiously, but with promptness and efficiency.

The State Board of Dental Examiners held their regular annual meeting at this time, when six candidates presented themselves for examination, after which the following persons being found qualified, received the certificate of the Board to that effect, viz: J. J. Stedman, M.D., Wellington, O.; Charles Elson, Wooster, O.; James L. Zell, Waynesville, O.

The other three applicants were laid over for further hearing and consideration.

The Board will hold another meeting on the first Wednesday of March, in the Dental College in Cincinnati.

### PORCELAIN DENTURES.

"What about mineral plates?" "How do dentures made wholly of porcelain, compare with other styles of work?" "Would you advise dentists generally to use it?"

These questions are so often asked that we have concluded to give expression to a few thoughts upon the subject. Like most other kinds of material and styles of dentures, the mineral plates, have their advantages and disadvantages.

By the use of this material in the construction of artificial teeth, nature's arrangement and beauty may be more nearly imitated than by any other, except continuous gum work; restoration of contour may be made quite as perfectly as by any other method.

When a plate of porcelain is well fitted to the parts upon which it is to be used, it is more comfortable to the wearer, than plate of any other material; its conduction of thermal change, is not objectionably plus nor minus. It irritates the mucous membrane less than any other material in common use.

It is especially valuable in peculiar cases in which almost everything else produces irritation. It is much lighter than continuous gum; and the same adaptation of the porcelain plate will insure greater firmness of attachment.

It has disadvantages which are serious obstacles to its general adoption by the profession. It requires a large amount of experience with manipulative skill of a high order, and great care to make it, and this is especially so with regard to its adaptation.

Another obstacle is found in the liability to breakage and almost impossibility of repair when broken; for this there is some compensation, in the facility with which subsequent pieces may be reproduced, by one entirely familiar with its working. In order to attain the best results, the workman should be almost in constant practice with it.

Those who have worn plates of other material and substituted this in almost every instance, give it the preference, as being more comfortable, less irritating and less liable to become foul than most other kinds of work.

It is rarely broken in use in the mouth, but generally when being handled out of the mouth, droping or striking it.

### VOLUME XXX—1876.

With this No. begins the thirtieth Vol. of the REGISTER.

It has passed through a period of twenty nine years, without a break or interruption in its issue; perhaps of no other dental journal can this be said.

The REGISTER has thus lived and been sustained, because it has always had in the profession many firm friends, who have manifested their friendship and regard, by deeds as well as words.

It has ever been the aim of the REGISTER to present correct principles, theory based upon truth.

By this we do not mean to say, however, that nothing, at variance with these, has ever appeared in the pages of the REGISTER; articles have sometimes appeared that have drawn forth sharp criticism, because of the errors they contained.

It is sometimes necessary to put error in a position where it may be most completely refuted.

Men oftentimes entertain false views and opinions, in sincerity and honesty, which subjected to the analysis of wholesome criticism are corrected or broken down.

The REGISTER will endeavor to present whatever shall best subserve the interest of the profession.

The control and direction of the journalistic and editorial department is wholly and absolutely in the hands of the editor, now may our readers one and all enjoy a happy and prosperous New Year.

### REPLACING EXTRACTED TEETH.

This subject is eliciting more attention now than ever before; and what will be the outcome is not easily decided now, nor is it very safe to predicate an opinion. There are a number of men in the profession who are experimenting in this direction, and some claim that they obtain very desirable results; in such cases for instance as the removal of diseased—even abscessed—teeth, freeing the root or roots from all diseased tissue or matter, then replacing; and in the great majority of such cases it is claimed that the roots will become attached and firmly fixed in the sockets, and be as serviceable as before they were diseased. It has for a long time been known that teeth free from disease, upon being extracted and returned to the sockets from whence they were taken, would again unite if returned in a short time. Teeth have united and become firm after having been out of their sockets for five hours. This is a mode of treatment which if applicable to diseased teeth, especially, is worthy of attention and experiment. We will have something further to say upon the subject ere long; in the mean time we hope to hear from those who are testing its practicability.

### THE LIST OF DENTISTS.

This list, prepared under the direction of the Ohio State Dental Society, was issued a short time since. This is a very important movement on the part of the profession, and the State Society especially.

It seemed impossible, in the time allowed to the work, to secure absolute accuracy in every particular, and even when it is made accurate it can not long remain so, for removals and changes will be continually occurring; but the names and professional standing can be correct. We would suggest that the revised edition contain an additional column, which shall give the number of years each dentist has been in practice, and also indicate by a * those who are members of the State Society.

Now it is very desirable that every dentist should co-operate in making this list as complete as possible. Let every one collect as much information as he can, and forward the same to Dr. J. M. Porter, of Toledo, or J. Taft, of Cincinnati.

Let every dentist make up a list of the dentists in his county including all the points above indicated, and forward it as early as possible.

### QUESTIONS ON THE STRUCTURE AND DEVELOPMENT OF THE HUMAN TEETH.

*For the Use of Dental Students, by C. L. Ford, M. D., Professor of Anatomy and Physiology in the University of Michigan.*

This is a new systematically arranged series of questions, upon the development and structure of the teeth and of the surrounding tissues, the object of this work, is to give the student definite points or landmarks along his course of study in this particular branch by which he may be more definitely guided.

The dental student in his curriculum of study finds embraced a large amount of reading, and if in college has many and varied subjects presented for his consideration in lectures and clinics, and it will generally be found, that the student fails to discriminate between the points and ideas of the greater importance and those of the lesser; such a guide as this little work, will relieve the student in a large measure from this embarassment.

This work will not only be valuable to the student, but to the practitioner, who desires to post himself in this direction, as well. It should be in the hands of every student and dentist.

The study of each branch would be made much more easy and rapid by having such a guide.

It has been prepared with special reference to the dental students, whom Prof. F. teaches, but it will be alike valuable to all others pursuing the same course.

Copies may be had by addressing Prof. C. L. Ford, Ann Arbor, Mich., or Editor the REGISTER, Cincinnati. Price 50c.

---

Three or four Editorials crowded out of this issue will appear in the February number.

